# Supplementary material for: A qualitative process evaluation within a clinical trial that used healthcare technologies for children with asthma–insights and implications
Source: PLoS One. 2023 Jan 5;18(1):e0280086. doi: 10.1371/journal.pone.0280086 (PMC9815588; doi:10.1371/journal.pone.0280086)
Supplement: S1 Appendix — (DOCX) [file pone.0280086.s001.docx]

**S1 Appendix. Participant (families) follow-up data collection procedure.**

1. Potential participants identified based on sampling criteria. Families were approached by research nurses at selected sites.
2. Potential participants who expressed an interest to participate (17) were followed up by two researchers on three separate occasions: from October 2019 – April 2020.
3. Six families were interviewed. The remainder of those who initially expressed an interest in taking part did not reply to further invitations (we did not notify prospective participants that they would not be interviewed).
