# Supplementary material for: A qualitative process evaluation within a clinical trial that used healthcare technologies for children with asthma–insights and implications
Source: PLoS One. 2023 Jan 5;18(1):e0280086. doi: 10.1371/journal.pone.0280086 (PMC9815588; doi:10.1371/journal.pone.0280086)
Supplement: Attachment — Submitted filename: Response to Reviewer.docx [file pone.0280086.s003.docx]

| Editor Comments | Authors’ Response |
| --- | --- |
| Thanks for the revisions, please address the final comment from R2: "the final sampling size with justification for selecting this size, in the section "Sampling and recruitment". | Thank you for the positive feedback on our manuscript. We have now included the final sample size in the “Sampling and recruitment” section of the methods (line 135, tracked changes – staff sample and line 155, tracked changes – family sample). The justifications for sampling size/method/criteria is also highlighted in the same section (lines 117-123, tracked changes – staff sample, and lines 154-157, tracked changes – family sample). This information was originally presented in the findings section. |
| Any changes to the reference list should be mentioned in the rebuttal letter that accompanies your revised manuscript. | We have changed the order of the references to accommodate for revised changes (as specified above) that included references. |
